# Interlaboratory Comparison Reveals State of the Art in Microplastic Detection and Quantification Methods

**DOI:** 10.1021/acs.analchem.4c05403

**Published:** 2025-04-17

**Authors:** Dmitri Ciornii, Vasile-Dan Hodoroaba, Nizar Benismail, Alina Maltseva, Juan F. Ferrer, Jiamin Wang, Raquel Parra, Ronan Jézéquel, Justine Receveur, Dina Gabriel, Andreas Scheitler, Christa van Oversteeg, Jorg Roosma, Alex van Renesse van Duivenbode, Tim Bulters, Michela Zanella, Alessandro Perini, Federico Benetti, Dora Mehn, Georg Dierkes, Michael Soll, Takahisa Ishimura, Marius Bednarz, Guyu Peng, Lars Hildebrandt, Mathias Peters, Seung-Kyu Kim, Jochen Türk, Felix Steinfeld, Jaehak Jung, Sanghee Hong, Eun-Ju Kim, Hye-Weon Yu, Sven Klockmann, Christoph Krafft, Julia Süssmann, Shan Zou, Alexandra ter Halle, Andrea M. Giovannozzi, Alessio Sacco, Marta Fadda, Mara Putzu, Dong-Hoon Im, Nontete Nhlapo, Priscilla Carrillo-Barragán, Natascha Schmidt, Dorte Herzke, Alessio Gomiero, Adrián Jaén-Gil, Damien J. E. Cabanes, Martin Doedt, Vitor Cardoso, Antje Schmitz, Moritz Hawly, Huajuan Mo, Justine Jacquin, Andy Mechlinski, Gbotemi A. Adediran, Jose Andrade, Soledad Muniategui-Lorenzo, Anja Ramsperger, Martin G. J. Löder, Christian Laforsch, Tanja Cirkovic Velickovic, Daniele Fabbri, Irene Coralli, Stefania Federici, Barbara M. Scholz-Böttcher, Jacopo la Nasa, Greta Biale, Cassandra Rauert, Elvis D. Okoffo, Anna Undas, Lihui An, Volker Wachtendorf, Petra Fengler, Korinna Altmann

**Affiliations:** † Bundesanstalt für Materialforschung und-prüfung (BAM), Unter den Eichen 87, 12205 Berlin, Germany; ‡ Nestlé Quality Assurance Center Vittel, 1020 Avenue Georges Clemenceau, 88804 Vittel Cedex, France; § AIMPLAS—Plastics Technology Centre, Gustave Eiffel 4, 46980 Paterna, Valencia, Spain; ∥ Beijing Academy of Science and Technology (Beijing Center for Physical and Chemical Analysis), No. 27, Xisanhuan (N) Rd, Haidian District Beijing, Beijing 100089, China; ⊥ CAPTOPLASTIC S.L., Calle de Génova, 11, 1°izda, Chamberí, 28004 Madrid, Spain; # Centre of Documentation, Research and Experimentation on Accidental Water Pollution, 715 Rue Alain Colas, CS 41836, 29218 Brest, France; ¶ Currenta GmbH & Co. OHG, Chempark Leverkusen, 51368 Leverkusen, Germany; ∇ DIL German Institute of Food Technology, Professor-von-Klitzing-Straße 7, 49610 Quakenbrück, Germany; ○ Rijkswaterstaat, Ministry of Infrastructure and Water Management, Zuiderwagenplein 2, 8224 AD Lelystad, The Netherlands; ⧫ TNO, Netherlands Organisation for Applied Scientific Research, Princetonlaan 6, 3584 CB Utrecht, The Netherlands; †† ECSIN-European Center for the Sustainable Impact of Nanotechnology—EcamRicert SRL, C.so Stati Uniti 4, 35127 Padova, Italy; ‡‡ European Commission—Joint Research Centre, Via E. Fermi, 2749, 21027 Ispra VA, Italy; §§ Bundesanstalt für Gewässerkunde, Am Mainzer Tor 1, 56068 Koblenz, Germany; ∥∥ Frontier Laboratories Europe, Bandstrasse 39B, 45359 Essen, Germany; ⊥⊥ Frontier Laboratories Ltd. 4-16-20, Saikon, Koriyama, Fukushima 963-8862, Japan; ## Umweltbundesamt, Corrensplatz 1, 14195 Berlin, Germany; ¶¶ Helmholtz Centre for Environmental Research—UFZ, Department of Environmental Analytical Chemistry, Permoserstrasse 15, 04318 Leipzig, Germany; ∇∇ Institute of Coastal Environmental Chemistry, Department for Inorganic Environmental Chemistry, Helmholtz-Zentrum Hereon, Max-Planck-Straße 1, 21502 Geesthacht, Germany; ○○ Hohenstein Laboratories GmbH & Co. KG, Schlosssteige 1, 74357 Boennigheim, Germany; ⧫⧫ Department of Marine Science, College of Natural Sciences, Incheon National University, 119 Academy-ro, Yeonsu-gu, Incheon 22012, Republic of Korea; ††† Institute for Energy and Environmental Technology e.V., Bliersheimer Street 58-60, 47229 Duisburg, Germany; ‡‡‡ RheinMain University of Applied Sciences, Faculty of Engineering, Institute for Environmental and Process Engineering, Am Brückweg 26, 65248 Rüsselsheim, Germany; §§§ Korea Institute of Analytical Science and Technology, SeoulSup AK Valley, Seongsuil-ro 99, Seongdong-gu, Seoul 04790, South Korea; ∥∥∥ Ecological Risk Research Division, South Sea Research Institute (SSRI), Korea Institute of Ocean Science and Technology (KIOST), 41 Jangmok-1Gi, Jangmok-myon, Geoje-Shi 656-834, Republic of Korea; ⊥⊥⊥ Department of Civil Engineering, Seoul National University of Science and Technology, Seoul 01811, Republic of Korea; ### K-water, Sintanjin-ro 200, Daedeok-gu, 34350 Daejeon, Republic of Korea; ¶¶¶ Labor IBEN GmbH, Am Lunedeich 157, 27572 Bremerhaven, Germany; ∇∇∇ Leibniz Institute of Photonic Technology e.V. (IPHT), Albert-Einstein-Straße 9, 07745 Jena, Germany; ○○○ Department of Safety and Quality of Milk and Fish Products, Max Rubner-Institut, Federal Research Institute of Nutrition and Food, Hermann-Weigmann-Straße 1, 24103 Kiel, Germany; ⧫⧫⧫ Metrology Research Centre, National Research Council Canada, 100 Sussex Drive, Ottawa, Ontario K1A 0R6, Canada; †††† Laboratoire Softmat, Université de Toulouse, CNRS, UMR 5623, Bâtiment 2R1, 118 Route de Narbonne, 31062 Toulouse Cedex 9, France; ‡‡‡‡ National Institute for Metrological Research, Strada delle Cacce, 91 10135 Torino, Italy; §§§§ Marine Environment Research Division, National Institute of Fisheries Science, Busan 46083, Republic of Korea; ∥∥∥∥ National Metrology Institute of South Africa, Private Bag X34, Lynnwood Ridge, Pretoria 0040, South Africa; ⊥⊥⊥⊥ The Dove Marine Laboratory, Newcastle University, Newcastle upon Tyne NE1 7RU, U.K.; #### NILU, Hjalmar Johansens Gate 14, 9007 Tromsø, Norway; ¶¶¶¶ Climate and Environment department, Norwegian Research Centre, Mekjarvik 12, 4072 Randaberg, Norway; ∇∇∇∇ Laboratoire Phytocontrol, 180 Rue Philippe Maupas, 30035, Nîmes, Francehytocontrol, Nîmes, France; ○○○○ Plastics Institute for Medium-sized Businesses, Karolinenstraße 8, 58507 Lüdenscheid, Germany; ⧫⧫⧫⧫ Direção de Laboratórios, Empresa Portuguesa das Águas Livres, S.A.—EPAL, 1800-031 Lisboa, Portugal; ††††† Private Diepholz University of Economics and Technology, Am Campus 2, 49356 Diepholz, Germany; ‡‡‡‡‡ SGS Institut Fresenius GmbH, Königsbrücker Landstraße 161, 01109 Dresden, Germany; §§§§§ SGS Testing & Control Services Singapore Pte Ltd, 30 Boon Lay Way No. 03-01, Singapore, 609957, Singapore; ∥∥∥∥∥ Biopôle Clermont-Limagne, Technical Center for Plastics Processing in France, 3 Rue Emile Duclaux, 63360 Saint-Beauzire, France; ⊥⊥⊥⊥⊥ PiCA Prüfinstitut Chemische Analytik GmbH, Rudower Chaussee 29, 12489 Berlin, Germany; ##### United Kingdom Centre for Ecology and Hydrology, Wallingford, Oxfordshire OX10 8BB, U.K.; ¶¶¶¶¶ Group of Applied Analytical Chemistry, Institute of Environmental Sciences (IUMA), Faculty of Sciences, University of A Coruña, Campus da Zapateira, 15071 A Coruña, Spain; ∇∇∇∇∇ Animal Ecology I and BayCEER, University of Bayreuth, Universitätsstraße 30, 95445 Bayreuth, Germany; ○○○○○ University of Belgrade-Faculty of Chemistry, Studentski trg 16, 11000 Belgrade, Serbia; ⧫⧫⧫⧫⧫ Department of Chemistry “Giacomo Ciamician”, University of Bologna, Technopole of Rimini, Via Dario Campana 71, 47922 Rimini, Italy; †††††† Department of Mechanical and Industrial Engineering & INSTM RU of Brescia, University of Brescia, Via Branze, 38 25123 Brescia, Italy; ‡‡‡‡‡‡ Institute for Chemistry and Biology of the Marine Environment, University of Oldenburg, Carl-von-Ossietzky-Straße 9-11, 26129 Oldenburg, Germany; §§§§§§ Department of Chemistry and Industrial Chemistry, University of Pisa, Via G. Moruzzi 13, 56124 Pisa, Italy; ∥∥∥∥∥∥ Queensland Alliance for Environmental Health Sciences, The University of Queensland, 20 Cornwall Street, Woolloongabba 4102, Queensland, Australia; ⊥⊥⊥⊥⊥⊥ Wageningen Food Safety Research, Part of Wageningen University & Research, 6708 WB Wageningen, The Netherlands; ###### State Key Laboratory of Environmental Criteria and Risk Assessment, Chinese Research Academy of Environmental Sciences, No. 8, Dayangfang, Beiyuan, Beijing 100012, China

## Abstract

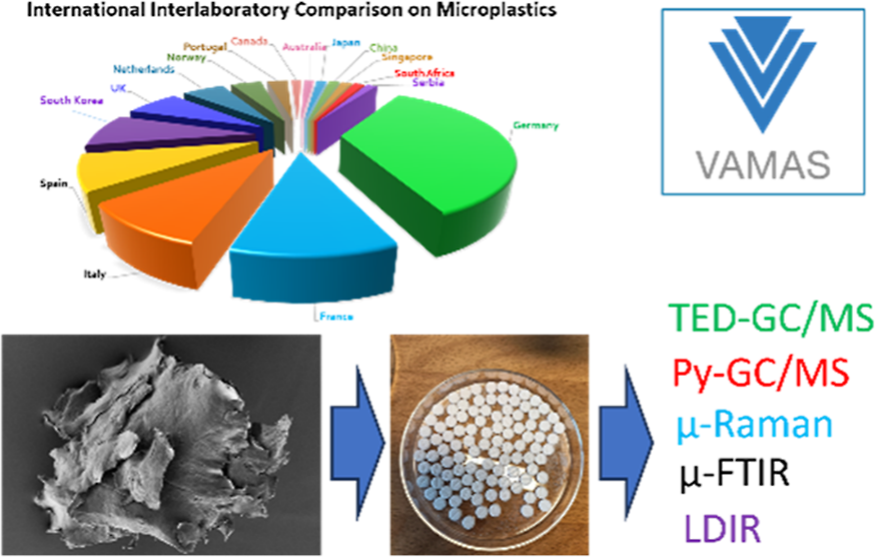

In this study, we investigate the current accuracy of widely used microplastic (MP) detection methods through an interlaboratory comparison (ILC) involving ISO-approved techniques. The ILC was organized under the prestandardization platform of VAMAS (Versailles Project on Advanced Materials and Standards) and gathered a large number (84) of analytical laboratories across the globe. The aim of this ILC was (i) to test and to compare two thermo-analytical and three spectroscopical methods with respect to their suitability to identify and quantify microplastics in a water-soluble matrix and (ii) to test the suitability of the microplastic test materials to be used in ILCs. Two reference materials (RMs), polyethylene terephthalate (PET) and polyethylene (PE) as powders with rough size ranges between 10 and 200 μm, were used to press tablets for the ILC. The following parameters had to be assessed: polymer identity, mass fraction, particle number concentration, and particle size distribution. The reproducibility, S_R_, in thermo-analytical experiments ranged from 62%–117% (for PE) and 45.9%–62% (for PET). In spectroscopical experiments, the S_R_ varied between 121% and 129% (for PE) and 64% and 70% (for PET). Tablet dissolution turned out to be a very challenging step and should be optimized. Based on the knowledge gained, development of guidance for improved tablet filtration is in progress. Further, in this study, we discuss the main sources of uncertainties that need to be considered and minimized for preparation of standardized protocols for future measurements with higher accuracy.

## 1

Microplastic pollution has become a matter of high concern in recent years and, despite being studied for about 20 years now,^1^ continues to hold a special place in political debates on all levels.^2^ To date, several prevention and monitoring measures from regulatory bodies have been undertaken. The first regulatory achievement was SB 1422: California Safe Drinking Water Act: microplastics, undertaken in 2018 and accompanied by a laboratory accreditation study.^3,4^ Another initiative was the Single-Use Plastics Directive of 2021^5^ which included a ban on marketing of drinking straws or nonreusable tableware within the EU. Moreover, monitoring initiative for MPs >20 μm was adopted within the Drinking Water Framework Directive.^6^ This was followed in April 2024 by the EU Urban Wastewater Directive (UWWTD).^7^ On January 1, 2025, the directive entered into force. The UWWTD does not explicitly outline specific methodologies for MP analysis; however, it requires member states to monitor water quality, develop and implement monitoring programs, and utilize the best available science and technology for analysis of micropollutants, to which MP also belongs. In March 2024, the European Commission adopted a decision on methodology to measure microplastics in water intended for human consumption.^8^

Data collected on microplastic load in the environment is often ambiguous, and transport pathways into water (limnic and marine ecosystems), air, and soil compartments are not well understood. Different analytical laboratories across the world are using different sampling protocols, sampling seasons, and sample preparation procedures to collect microplastics, etc. Similarly, analysis of microplastic samples occurs with different analytical methods and instrumental setups, e.g., size threshold, leading to poorly comparable results. Currently, there is an urgent need for harmonization of methodology and standard operating procedures (SOPs) as well as the elaboration of minimum requirements for profound data.

Parameters to be assessed during microplastic analysis are specified in ISO 24187:2023, where “The amount of microplastics in a given matrix can be measured in different ways, i.e., as the number (of particles) or mass content/fraction in relation to the sample’s quantity, which itself can be based on various units (volume, weight, etc.)”.^9^ Based on this, traditional microplastic analysis includes polymer type identification, particle number concentration, size distribution, and particle mass in a sample. Many methods have been successfully used in microplastic analysis so far;^10−14^ however, most used methods for particle number counting and polymer identity are micro-Fourier-transform infrared spectroscopy (μ-FTIR)^15−19^ and micro-Raman spectroscopy (μ-Raman).^20−22^ Lowest limits for identification, depending on the instrument, lie within 10–20 μm (for μ-FTIR spectroscopy) and around 0.5–5 μm (for μ-Raman spectroscopy). The lower limit of identification for the μ-Raman technique is due to a laser of a short wavelength used in this technique, whereas in μ-FTIR spectroscopy, an infrared source is used. Therefore, with μ-Raman spectroscopy in the imaging mode, higher spatial resolution can be obtained than with μ-FTIR spectroscopy in the imaging mode. For mass content and polymer identity, the most common methods are pyrolysis gas chromatography mass spectrometry (Py-GC/MS),^23−27^ and thermal extraction desorption gas chromatography mass spectrometry (TED-GC/MS).^28−31^ In this ILC, the four above-mentioned methods were employed: μ-FTIR and μ-Raman spectroscopies, Py-GC/MS, and TED-GC/MS. Additionally, a variety of infrared spectroscopy, quantum-cascade laser infrared reflectance spectrometry (QCL-IRRAS), also known under the commercial acronym as Laser Direct Infrared (LDIR) spectroscopy, has been used by some participants. Since this name is more recognizable, we will use this acronym.

Thermo-analytical methods are generally less time-consuming in terms of sample preparation, but experiments may take up to 1 h per sample. Shortcomings in thermoanalytics are the limit of detection, no analysis of a single particle, sample destruction, and no information on the size and shape of particles. Spectroscopical techniques are often more time-consuming due to the intense need for sample preparation and measurement times but are nondestructive, and, when applied in imaging mode, can give information about the size and shape of a single particle.

Despite a variety of available methods and many decades of development in microplastic analytics, reliable microplastic analysis remains a very difficult task and requires various microplastic (certified) reference materials (CRMs), extensive funding, and laboratories with proven expertise in the field. With the provided set of microplastic RMs for the present ILC, operating protocols for tablet filtration, measurement, and data reporting, and with the use of a variety of analytical techniques by over 50 laboratories worldwide, we expected valuable statistical data. The obtained results as well as test materials will be transferred into the ISO standardization body ISO/TC 147/SC 2 to the documents ISO/DIS 16094-2 and ISO/DIS 16094-3, helping to create standards and achieve comparable results worldwide.

**Figure 1:**
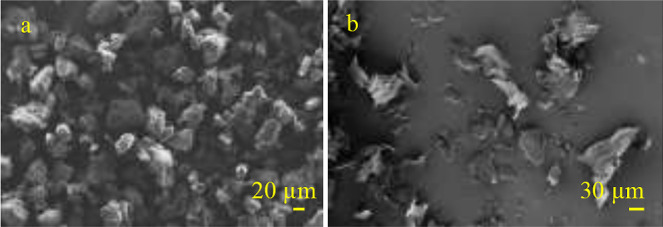
SEM micrographs of microplastic particles (a) PET and (b) aged PE.

To date, many ILCs on the analysis of microplastics have been carried out (Table S1, Supporting Information), creating a solid basis and delivering valuable findings but also revealing challenges in method harmonization and microplastics RM development. In the present study, evaluation of particles in a smaller size range is addressed: 1–100 μm for PET and 1–250 μm for aged PE films (Figure 1). To resemble environmental MP samples and mimic an aged PE material, the PE film was preweathered. Furthermore, microplastic samples were prepared as RMs embedded in a food matrix (to be possibly considered as a suitable RM for drinking water framework directive monitoring).

## 2 Experimental Section

### 2.1 Preparation of Microplastic Materials

For this ILC, BAM (Bundesanstalt für Materialforschung und -prüfung) provided microplastic RMs, PET, and aged PE. Further information on preparation is available in the BAM webshop (https://webshop.bam.de/webshop_en/reference-material/polymeric-materials/microplastics.html). The property of interest for both RMs is the *D*_50_ value (*D*_50_ value describes the particle diameter of the measured volume below which 50% of particles fall). The shape of microplastic powders was assessed by means of scanning electron microscopy (SEM), see Figure 1. PET RM had a nominal *D*_50_ value of 42.45 ± 0.17 μm, and aged PE had a nominal *D*_50_ value of 61.18 ± 1.30 μm, see Figure S2. *D*_50_ values were determined by means of a laser diffraction system (Sympatec, Clausthal-Zellerfeld, Germany) consisting of a RODOS-L dry dispersion unit, an ASPIROS-L micro dosing unit, and a HELOS-BR sensor.

The RMs used and their properties are summarized in Table 1.

**Table 1:** Summary of Properties of Microplastic Materials Used in the ILC

	polymer #1	polymer #2
polymer	PET	PE
*D*_50_ (μm)	42.45	61.18
pristine polymer density (g/cm^3^)	1.38	0.93–0.97
particle shape	irregular	irregular
starting material	PET granulate	PE film

To achieve an easy way of transportation of microplastic polymers, both obtained microplastic powders (either PET or aged PE) were mixed with a water-soluble matrix containing polyethylene glycol (6.4% w/w) and lactose (93.3% w/w) and were pressed to tablets with a defined mass of polymer (see Figure S4). Tablets were pressed with a dedicated press machine (TDP 5 Desktop Tablet Press, LFA Machines, UK) at a 50 kN pressure. The hardness of tablets was adjusted such that they dissolved quickly but withstood physical stress during transportation. The tablets’ weight was 250 ± 5 mg (Mettler Toledo AT DeltaRange analytical balance), and tablets, varying by more than ±5 mg, were discarded. The expected polymer mass per tablet was empirically calculated from the mass of the polymer (weighed mass for approximately 1000 tablets) in a defined mass of matrix. Assuming a ±5 mg tablet mass uncertainty, the mass fraction of PET/tablets for thermo-analytical experiments was expected to be 556 ± 11.12 μg (equivalent to 2.22 ± 0.04 μg/mg) and that of aged PE: 265 ± 5.3 μg (equivalent to 1.06 ± 0.02 μg/mg). To simplify counting of particles, tablets for spectroscopic methods contained a reduced amount of the polymer: either 27.6 ± 0.55 μg of PET (equivalent to 0.11 μg/mg) or 17.6 ± 0.35 μg of aged PE (equivalent to 0.07 μg/mg). All participants were provided with 6 tablets containing aged PE, 6 tablets containing PET, and 6 blank samples (not containing the polymer of interest) along with an SOP to prepare polymer mixed samples. Participants working with thermo-analytical methods were provided with an additional 1 g of pure aged PE and PET polymer for calibration purposes.

### 2.2 Homogeneity Studies

An internal homogeneity study was performed to ensure that tablets contained a desired amount (mass fraction and particle number concentration) of polymer microparticles. For a homogeneity study regarding mass fraction, a batch of 19 tablets containing one polymer type was measured with two thermo-analytical methods, i.e., TED-GC/MS and thermogravimetric analysis (TGA). Prior to measurements, polymer tablets were dissolved with Milli-Q water and filtered (see Figure S5) according to a filtration protocol developed at BAM. Tablets were filtered in a special crucible (see Figure S5a).^32^

Regarding the particle number concentration, a batch of 20 tablets containing one polymer type were analyzed with SEM by counting all particles left on the substrate (CSP Si-filter, 9 μm pores, SmartMembranes GmbH) after a tablet filtration process. In our case, samples did not contain any intentionally added adventitious particles but solely lactose and PEG, which are both well soluble in water. Therefore, assuming that only plastic particles were left on the filter after filtration, we counted polymer particles based on their shape and corresponding contrast in electron micrographs. Particle size distribution was determined by using ImageJ^33^ software by measuring Feret_max_ values, with particle counting being performed manually.

Besides the studies conducted internally, we investigated the homogeneity of the microplastic tablets with respect to the particle number. An accredited institution supported us with a set of data on microplastic particle numbers in tablets (μ-FTIR and μ-Raman data). All samples were prepared under the same conditions and on same day according to a previously validated method, in agreement with Schymanski publication requirements and precautions^34^ and in agreement with ISO CD 16094-2.^35^ After the addition of 1 L of pure water on tablets for reconstitution, samples were passed through silicon filters (5 μm pore size). Once dried, the filters were analyzed with μ-FTIR (Thermo Nicolet iN10) and Raman (HORIBA Labram HR evolution) systems. The whole surface of the filter was screened, and each particle was analyzed for identification. Two blank tablets and one blank water were filtered and analyzed the same day, following exactly the same steps and the same process as with all samples. The blank water sample demonstrated good results, inferior to the limit of reporting of the laboratory, allowing validation of a series of samples. Raw data were categorized by the type of polymers and size class (Feret_max_) without any blank subtraction.

## 3 Results

### 3.1 Homogeneity Studies

Results obtained in thermo-analytical experiments expressed as μg polymer per mg tablet varied in an acceptable range (see Table S2). Results on the particle number carried out by an accredited laboratory with both methods, μ-FTIR and μ-Raman spectroscopies, are summarized in Tables S3 and S4, respectively.

SEM is a powerful method for visualization of small particles down to several nm; however, manual counting of hundreds/thousands of particles is quite a challenging task. Particles <10 μm could hardly be assigned due to poor contrast and slurry particle boundaries. The drawback of SEM analysis is, furthermore, that the chemical identity of the material remains uncertain. Besides, agglomerates of particles can sometimes provide difficulties in size assessment. Table S5 summarizes results from SEM experiments. A direct comparison of the results measured with different methods is a challenging task without knowing the “true” values of mass fraction and particle numbers. For this reason, we used data from a homogeneity study conducted at BAM (for thermo-analytical methods) and from a homogeneity study for spectroscopical methods as reference values for the mass fraction (see Table S2) and particle number (see Tables S3 and S4).

### 3.2 Data Evaluation

**Figure 2:**
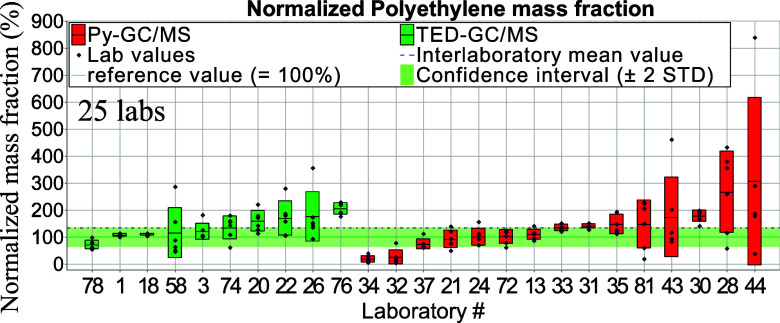
Normalized aged PE mass fraction. Red and green bars—standard deviations of intralaboratory results (repeatability).

Out of 84 registered laboratories, only 56 reported their results. Results obtained from ILC participants were evaluated at BAM using software ProLab Standard^36^ that works in compliance with ISO 5725-2:2019 Accuracy (trueness and precision) of measurement methods and results.^37^ Data from each laboratory are presented as bar plots. All values obtained from ILC participants were normalized to reference values; i.e., each value was divided by the reference value and multiplied by 100. In the following calculation, we will explain data treatment on a concrete example:

Reference values for aged PE and PET as obtained from the BAM homogeneity study: PE → 0.83 ± 0.15 μg/mg. These are absolute values. We then divided them by themselves (we normalized them against themselves) to obtain PE → 1 ± 0.1807. To represent this as “%”, we multiplied everything by 100%: PE → 100% ± 18.07%. So, in all graphs, the reader will find the reference value at 100% (the *y*-axis is represented here always in “%”). The confidence interval shows the range between ±2 standard deviations corresponding to a reference value. For our example, it means that the confidence interval for PE = ± 2(18.07%) = ± 36.14%; i.e., on the graph, it will appear as a filled region of width 63.88% to 136.14%. The same calculation applies for PET. A participant X delivers absolute values v1–v6: 0.951, 0.927, 0.920, 0.850, 0.841, and 0.897 (μg/mg), respectively. We divide these by 0.83 μg/mg to obtain 1.144, 1.114, 1.106, 1.022, 1.011, and 1.078 and then multiply them by 100% → 114.4%, 111.4%, 110.6%, 102.2%, 101.1%, and 107.8%, respectively. These values are then plotted in the graph (see Figure 2, Lab 1).
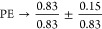


### 3.3 ILC Results from Thermo-analytical Methods

**Figure 3:**
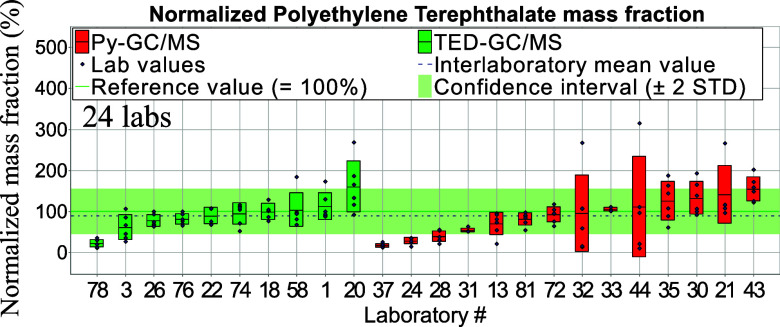
Normalized PET mass fraction. Red and green bars—standard deviations of intralaboratory results (repeatability).

Data from both thermo-analytical methods (Py-GC/MS and TED-GC/MS) were normalized against reference values obtained from TED-GC/MS results (homogeneity study conducted at BAM, see Table S2). For both thermo-analytical methods, Py-GC/MS and TED-GC/MS, the confidence interval refers to TED-GC/MS data from the homogeneity study since reference values for Py-GC/MS were not measured. Table 2 shows mass fractions in absolute values. It is noticeable that both thermo-analytical methods are in high agreement with each other (see Table 2) regarding mean values for both polymer types. Figures 2 and 3 display normalized mass fraction values.

**Table 2:** Mass Fraction for Both Polymers and Both Methods (in μg/mg)

	TED-GC/MS (μg/mg)	Py-GC/MS (μg/mg)
PET	1.69 ± 0.67	1.67 ± 0.81
aged PE	1.14 ± 0.33	1.11 ± 0.64

The relative standard deviations (RSD) for PET were below 30% for both methods, except for particles >500 μm, which could be agglomerates. In contrast, RSD values for aged PE were higher, probably due to irregular shape and possible sticking between PE fragments.

All participants correctly identified both polymer types. It is worth mentioning that three laboratories reported relative standard deviations as low as ∼4%, and three other laboratories reported >100%. Many participants reported saturation of the detector when measuring PET (default mass content: 556 μg per tablet). To circumvent this issue, some participants cut the filters into smaller pieces and measured them one by one and/or elevated the split ratio of the injector extremely. This point needs to be considered in future ILCs.

### 3.4 ILC Results from Spectroscopical Methods

For determination of particle size distribution, participants were asked to group particles according to the longest distance (maximum Feret diameter) into following categories, as required by ISO 24187:2023 “Principles for the analysis of microplastics present in the environment”: (1) 500–100 μm, (2) 100–50 μm, (3) 50–10 μm, (4) 10–5 μm, and (5) 5–1 μm.^38^ We decided to include an additional size class “>500 μm” to check how efficient the sieving process was (during preparation of microplastic powder) and whether possible agglomerates were formed.

#### 3.4.1 Total Particle Numbers per Tablet

**Figure 4:**
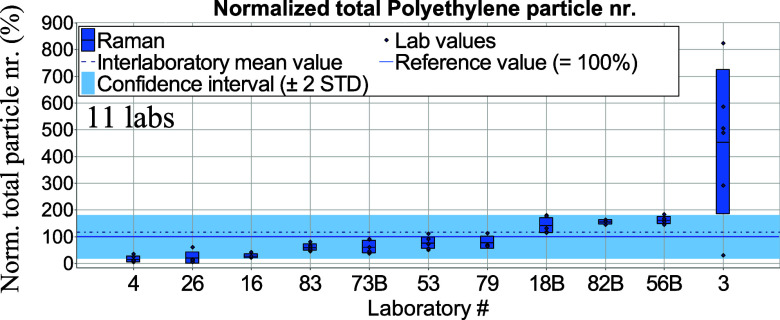
Total normalized aged PE particle numbers per tablet derived from μ-Raman spectroscopy measurements. Blue bars—standard deviations of intralaboratory results (repeatability).

**Figure 5:**
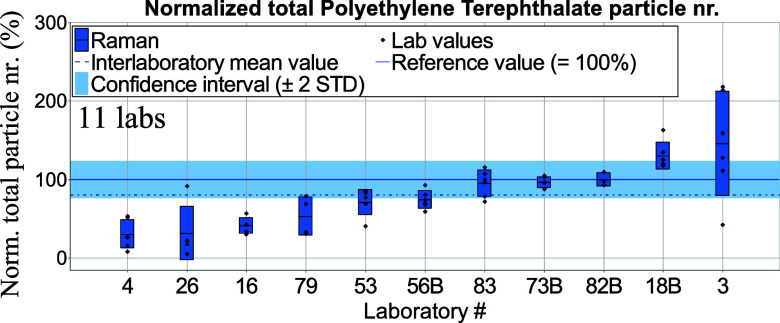
Total normalized PET particle numbers per tablet derived from μ-Raman spectroscopy measurements. Blue bars—standard deviations of intralaboratory results (repeatability).

**Figure 6:**
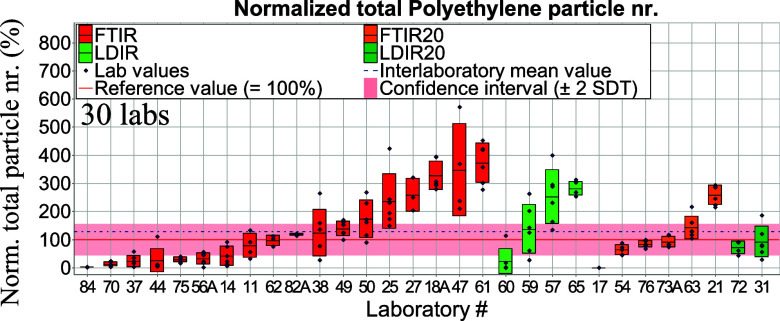
Total normalized aged PE particle numbers per tablet derived from μ-FTIR and LDIR spectroscopy measurements. Red, green, orange, and dark-green bars—standard deviations of intralaboratory results (repeatability).

**Figure 7:**
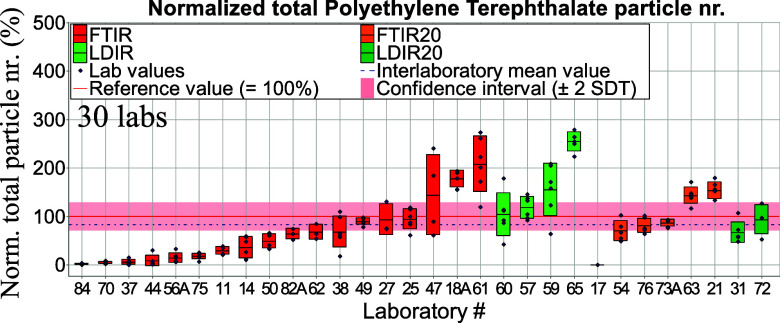
Total normalized PET particle numbers per tablet derived from μ-FTIR and LDIR spectroscopy measurements. Red, green, orange, and dark-green bars—standard deviations of intralaboratory results (repeatability).

Whereas μ-Raman spectroscopy theoretically allows particle dimension measurement down to 0.5–5 μm, IR spectroscopy is generally not so sensitive, and its limit of reliable detection often, but not always, lies above 20 μm. Due to differences in the limit of detection, we decided to display results for total particle number separately for μ-Raman spectroscopy (Figures 4 and 5) and in a combined form for μ-FTIR and LDIR spectroscopies (Figures 6 and 7) since the latter two methods are variations of IR spectroscopy.

μ-Raman spectroscopy results were normalized against reference values obtained from μ-Raman spectroscopy measurements (see Table S4). In the following graphs, letters “A” and “B” in the *X*-axis “Laboratory #” denote μ-FTIR and μ-Raman spectroscopy, respectively. Noticeably, many laboratories using the μ-Raman spectroscopy technique achieved results lying within the confidence interval (see Figure 4). Some participants, however, reported that they did not measure the whole filter (results were extrapolated). Data from μ-FTIR and LDIR spectroscopies were normalized against reference values obtained from the μ-FTIR homogeneity study; see Table S3. The confidence interval refers here to μ-FTIR data (Table S3). Figures 6 and 7 display total particle numbers obtained from μ-FTIR and LDIR spectroscopy as measured for aged PE and PET, respectively. Due to different instrument abilities among IR users, we decided to group results as follows: (i) μ-FTIR and LDIR referring to those who measured >10 μm and (ii) FTIR20 and LDIR20, referring to those who measured >20 μm.

#### 3.4.2 Particle Numbers in the Size Class >500 μm

Whereas total particle number and size class 10–50 μm are two parameters affected by different detection limits of the methods, other size classes, like >500 μm, 100–500 μm, and 50–100 μm are not, and therefore, we can compare corresponding results measured with different methods within these individual size classes. In the following, we will show results from these size classes as a comparison between all three methods, μ-Raman, μ-FTIR, and LDIR spectroscopies, all in the same plot (see Supporting Information, Figures S6 and S7).

The last step in the microplastic powder preparation procedure is sieving through a 500 μm mesh; therefore, it is expected that particles larger than 500 μm should not be present in tablets. Most laboratories did not detect any particles larger than 500 μm. However, some laboratories indeed measured such particles with higher numbers for aged PE than for PET, this being valid for both methods, μ-Raman spectroscopy and μ-FTIR spectroscopy, as presented in Figures S6 and S7. Participants using the LDIR method either did not report any particles >500 μm or just one particle per tablet. This could also be due to the automatic settings of the analysis limit on LDIR instruments, which is often restricted to a maximum of 500 μm (unless the operator changes the analysis limit manually). However, the presence of such large particles could, on the other hand, be explained by agglomeration of particles or if the longest dimension of particles was larger than 500 μm and other dimensions smaller so that it could pass through a 500 μm sieve during material production.

#### 3.4.3 Particle Numbers in the Size Class of 100–500 μm

In the particle size class of 100–500 μm, a large variation among results was noticeable. Some participants reported zero particles, and others several hundred particles for both techniques and both polymer types. Variations in the results obtained with LDIR spectroscopy were small. Independent of the measurement method used, values are mostly underestimated compared to reference values, which applies for both polymers, see Figures S8 and S9. Another observation made here was that mean values (for both polymers) deviated a lot from reference values.

#### 3.4.4 Particle Numbers in the Size Class of 50–100 μm

Here, a similar behavior as in the size class 100–500 μm could be observed. Some ILC participants reported no particles or very small numbers, whereas others reported over 500 particles. Values derived from μ-Raman and LDIR spectroscopies were mostly within the confidence interval (especially in case of aged PE), whereas μ-FTIR spectroscopy values were more heterogeneous among laboratories. In Figures S10 and S11, results are presented in a normalized form.

#### 3.4.5 Particle Numbers in the Size Range of 10–50 μm

As already mentioned, in this size class, we cannot directly compare results derived from μ-Raman spectroscopy and μ-FTIR spectroscopy due to limitations of the μ-FTIR spectroscopy method. Therefore, also here, we also show the results for μ-Raman spectroscopy separately (Figures S12 and S13) and in combined form for μ-FTIR and LDIR spectroscopies (Figures S14 and S15). From μ-Raman spectroscopy experiments, particularly in the case of aged PE, it can be noticed that, with few exceptions, values were within or close to the confidence interval, whereas in the case of PET, values scattered more obviously. However, interlaboratory mean values, both in case of aged PE and PET, lay quite close to reference values (see Figures S12 and S13).

Like in case of total particle numbers (Figures 6 and 7), we decided to split μ-FTIR and LDIR spectroscopy results (from size class 10–50 μm) in two groups: (i) FTIR and LDIR where participants measured >10 μm and (ii) FTIR20 and LDIR20 where participants measured >20 μm. It is noticeable that laboratories measuring in the range of 20–50 μm (both LDIR and μ-FTIR) slightly exceed reference values (Figures S14 and S15). This behavior applies to both polymers. In case of μ-FTIR and LDIR spectroscopy (results obtained for 10–50 μm), values also exceeded reference values.

#### 3.4.6 Particle Numbers in the Size Class of 5–10 μm

Since for this size class no reference values for μ-FTIR were available and only a few laboratories reported data on this size class, we only show results obtained from μ-Raman spectroscopy users, Figures S16 and S17. For both polymers, values were underestimated compared to reference values.

### 3.5 Other Polymers in Sample Tablets

Purity of the samples must be assessed. Participants reported identification of particles that could not be identified as aged PE or PET. Such noninterest polymer types in sample tablets were PBT, PP, PS, EVOH, EVAc, ABS, PMMA, PA, PU, PAN, PVC, PTFE, ABS, EVA, PC, PM, POM, PU, rubber, VDC, and PVA, pointing toward contamination. This should be addressed in future ILCs.

### 3.6 Blank Sample Tablets

In addition to two polymer types, aged PE and PET, which were found in blank samples, albeit in small number concentrations, other polymer types were also detected by some laboratories, e.g., PMMA, acrylate, urethane, PP, PVC, as well as some nonplastic particles like cellulose, protein, plant fibers etc. Unfortunately, data on background contamination levels from each participating laboratory were not collected. We will incorporate this as a learning point for future ILCs. Despite this limitation, the matrix material employed in this ILC proved to be suitable for testing representative microplastic materials.

## 4 Discussion

### 4.1 Main Sources of Uncertainties

To fully understand the results, it is essential to identify and discuss potential sources of measurement uncertainty. While this study highlights variability between laboratories (as indicated by standard deviations from repeated measurements), participants were not asked to quantify absolute measurement uncertainties. A systematic evaluation of these uncertainties is particularly challenging, especially when the “true” values remain unknown.

Table S6 summarizes the most significant sources of uncertainty in our opinion. The total uncertainty budget could consist of (but is not limited to) the following factors.

#### 4.1.1 Uncertainties Emerging from Properties of Representative Test Materials

i Sample homogeneity can be determined with the help of RSD. For the mass fraction of the polymer, a RSD of 27% was calculated from data obtained with TED-GC/MS, which represents a rather high value. Regarding the total particle number, RSDs were 14–28% for FTIR spectroscopy and 12–40% for μ-Raman spectroscopy.
ii The tablet mass was set at 250 mg with a variation of ±5 mg and therefore constitutes only a small contribution of 2% to the overall uncertainty.
iii Blank samples. Blank samples were found to contain polymers of interest, even though to a minor extent. However, background contamination was not asked to be assessed.

#### 4.1.2 Uncertainties Emerging from Microparticle Preparation for the Measurement

iv Another, yet undetermined, uncertainty source is the tablet filtration step. It is unknown how many particles are lost during such a process; each operator has a different filtration setup, filter, and working procedure. Here, most probably, rather smaller particles might get “lost” during filtration.

#### 4.1.3 Uncertainties Emerging from Methods and Instruments

v Contribution of method sensitivity is difficult to assess. Given that μ-Raman spectroscopy has a limit of detection down to a 1–5 μm range and μ-FTIR a 10–20 μm range, we can estimate that for some instruments, but not always, μ-FTIR spectroscopy “misses” particles <10 μm. This missing fraction constitutes about 4–5% of the total particle number—according to particle size distribution measurements, see Figure S2. These assumptions explain the lower particle numbers reported. However, higher particle numbers cannot be explained by a low method sensitivity.
vi Instrument calibration could account for a rather small contribution to uncertainty; however, this information is lacking and needs to be individually addressed in future ILCs.
vii Spectral library used and its accuracy.
viii Instrumental settings were recommended in the SOP distributed to all ILC participants; however, participants were free to use settings known to work reliably for their laboratory. All changes were reported in a dedicated Excel reporting sheet. For example, setting of threshold size or matching a spectrum against a spectrum in a spectral library could influence results a lot. However, we could not find any correlation between instrumental settings and results, but we assume that uncertainties might be in the middle range of significance.
ix Precision of the software used.

#### 4.1.4 Uncertainties Emerging from the Operator’s Work

x Measurement repeatability can be evaluated, representing precision within a laboratory by measuring *n* times (*n* = 6 in this ILC) the same test material but different samples. Some laboratories reported very good repeatability; however, some others reported high variations between 6 samples.
xi Operator’s accuracy.
xii Extrapolation of results.

We assume that laboratories work in a clean environment, but in future ILCs, background contamination should be assessed, as well.

### 4.2 Tablet Dissolution

It is worth mentioning that the filtration of tablets represents one of the most critical steps. At BAM, we have developed a filtration procedure which is functional; however, the loss of microplastic particles during filtration seems inevitable. It needs to be mentioned here that the exact filtration setup and instrumentation used to develop the filtration protocol at BAM might be completely different from the setup used at other laboratories. Some participants, for example, observed particles sticking to funnel walls. This might be one of the reasons for the large variation in particle number concentrations. The reason for this could be incomplete dissolution of tablets and formation of agglomerates, which could explain the presence of large unexpected “particles”. We received feedback that tablet dissolution did not always occur as expected, and this is why the SOP was not always followed. Some participants added either larger Milli-Q water volumes, warmed them up, held the tablet for longer timeframes in Milli-Q water, or shook beakers with the tablet inside. There is obviously much room for improvement.

### 4.3 Used Software for Analysis

According to some feedback from participants, another reason for the high discrepancy between spectroscopical methods might lie in the use of different software and databases. While for LDIR spectroscopy, most often used spectra interpretation software is Microplastic Starter 1, the software used for μ-Raman and μ-FTIR spectroscopies is often siMPle or PMF (Purency Microplastic Finder). PMF generally tends to overestimate detection of MPs as compared to siMPle. This fact might explain an overall good agreement among LDIR results and, on the other hand, high variation between μ-Raman and μ-FTIR spectroscopies.

### 4.4 Correlations

To better understand why some participants reported extremely high values (either in thermoanalysis or vibrational spectroscopy methods), we searched for correlations. We compared results from the group that reported the lowest values with the group that reported the highest values. We tried to correlate results with all possible factors, such as the method used, instrumental settings, laboratory experience, filters used, detection limit of the instrument (for vibrational methods), etc. We also compared results from automated counting approaches vs manual counting, in the hope of finding some sort of correlation to particle numbers. To our surprise, we could not identify any correlation of the reported results with anything. This leads us to the conclusion that discrepancies lie somewhere else and that we do not know if instruments, methods, or experience play a significant role in the obtained results or a combination thereof. The only observation which showed a certain degree of correlation was that more experienced laboratories proved smaller standard deviations; however, there was no clear direct correlation between experience and mean values reported.

## 5 Conclusions

In this study, we evaluated results as mean values of each laboratory with corresponding standard deviations based on statistics and repeatability. Mass is an important monitoring parameter and may be used for estimation of the occurrence of microplastics in the environment. However, to obtain a comprehensive picture of the occurrence, parameters such as particle number, size distribution, and shape are also crucial. The particles’ shape can be easily assessed by means of SEM measurements. Here it has to be stressed that particle shape assessment by means of SEM is only feasible if the particles’ identity is known. In a real environmental sample, such conditions are not given and SEM assessment is not meaningful. Accurate measurement of particle number concentration represents a huge challenge since it is very difficult to count all particle sizes. Dissolution of tablets represents a huge bottleneck for SEM analysis, being the most important step in the preparation of particles for measurement. Only a complete dissolution of tablets can guarantee that no other particles, except polymer particles, are left on the filter. Unfortunately, many participants reported incomplete tablet dissolution, and we assume that this might be the highest impact on variation in particle numbers reported by ILC participants. The lesson learned here is that more easily dissolvable tablets should be used.

Besides, as discussed above, it is important to consider the software and database used for the analysis of particle number as one of a variety of factors.

Today, microplastic analysis is still in its infancy, despite numerous studies on the topic, including the present ILC. Currently, key directions in this field are being established, methods are being validated, and reference materials (RMs) are under development. This process may take another 10 years to fully mature. However, with this study, we have moved closer to understanding major challenges, such as sources of measurement uncertainties in microplastic analysis. This issue must be explicitly addressed in future ILCs.

Real progress can only be made by investigating true values, which is achievable either by using certified reference materials (CRMs) or by assessing a comprehensive uncertainty budget. Similar challenges are likely to arise in the next related analytical task: the accurate measurement of nanoplastics. This study also serves as a strong foundation for future research on nanoplastics.

## Supporting Information Available

Additional experimental details and methods, including photographs of the experimental setup and charts of particle number for different size classes (>500 μm, 100–500 μm, 50–100 μm, 10–50 μm, and 5–10 μm) and particle size distribution, previous ILCs, table with uncertainty sources, and results from homogeneity studies in tabular (PDF)
