# Supplementary material for: Interlaboratory Comparison Reveals State of the Art in Microplastic Detection and Quantification Methods
Source: Anal Chem. 2025 Apr 17;97(16):8719–28. doi: 10.1021/acs.analchem.4c05403 (PMC12044667; doi:10.1021/acs.analchem.4c05403)
Supplement: Supplementary file 1 — ac4c05403_si_001.pdf [file ac4c05403_si_001.pdf]

Supporting Information

Interlaboratory comparison reveals state-of-the-art in microplastic detection and quantification methods

Dmitri Ciornii[1,*], Vasile-Dan Hodoroaba[1,*], Nizar Benismail[2], Alina Maltseva[2], Juan F. Ferrer[3], Jiamin Wang[4], Raquel Parra[5], Ronan Jézéquel[6], Justine Receveur[16], Dina Gabriel[7], Andreas Scheitler[8], Christa van Oversteeg[9], Jorg Roosma[10], Alex van Renesse van Duivenbode[10], Tim Bulters[10], Michela Zanella[11], Alessandro Perini[11], Federico Benetti[11], Dora Mehn[12], Georg Dierkes[13], Michael Soll[14], Takahisa Ishimura[15], Marius Bednarz[16], Guyu Peng[17], Lars Hildebrandt[18], Mathias Peters[19], Seung-Kyu Kim[20], Jochen Türk[21], Felix Steinfeld[22], Jaehak Jung[23], Sanghee Hong[24], Eun-Ju Kim[25], Hye-Weon Yu[26], Sven Klockmann[27], Christoph Krafft[28], Julia Süssmann[29], Shan Zou[30], Alexandra ter Halle[31], Andrea M. Giovannozzi[32], Alessio Sacco[32], Marta Fadda[32], Mara Putzu[32], Dong-Hoon Im[33], Nontete Nhlapo[34], Priscilla Carrillo-Barragán[35], Natascha Schmidt[36], Dorte Herzke[36], Alessio Gomiero[37], Adrián Jaén-Gil[37], Damien J.E. Cabanes[38], Martin Doedt[39], Vitor Cardoso[40], Antje Schmitz[41], Moritz Hawly[42], Huajuan Mo[43], Justine Jacquin[44], Andy Mechlinski[45], Gbotemi A. Adediran[46], Jose Andrade[47], Soledad Muniategui-Lorenzo[47], Anja Ramsperger[48], Martin G. J. Löder[48], Christian Laforsch[48], Tanja Cirkovic Velickovic[49], Daniele Fabbri[50], Irene Coralli[50], Stefania Federici[51], Barbara M. Scholz-Böttcher[52], Jacopo la Nasa[53], Greta Biale[53], Cassandra Rauert[54], Elvis D. Okoffo[54], Anna Undas[55], Lihui AN[56], Volker Wachtendorf[1], Petra Fengler[1], Korinna Altmann[1,*]

[1]Bundesanstalt für Materialforschung und – prüfung (BAM), Unter den Eichen 87, 12205 Berlin, Germany; E-Mail: dmitri.ciornii@bam.de; dan.hodoroaba@bam.de; korinna.altmann@bam.de

[2]Nestlé Quality Assurance Center Vittel (NQAC Vittel), 1020 Avenue Georges Clemenceau - 88804 Vittel Cedex, France

[3]AIMPLAS – PLASTICS TECHNOLOGY CENTRE, Gustave Eiffel 4, 46980 Paterna, Valencia, Spain

[4]Beijing Academy of Science and Technology (Beijing Center for Physical and Chemical Analysis), No.27, Xisanhuan (N) Rd., Haidian Dist. Beijing, Beijing, 100089 China

[5]CAPTOPLASTIC S.L., Calle de Génova, 11, 1ºizda, Chamberí, 28004 Madrid, Spain

[6]Centre of Documentation, Research and Experimentation on Accidental Water Pollution, 715 rue Alain Colas, CS 41836, 29218 Brest, France

[7]Currenta GmbH & Co. OHG, Chempark Leverkusen, 51368 Leverkusen, Germany

[8]DIL German Institute of Food Technology, Professor-von-Klitzing-Straße 7, 49610 Quakenbrück, Germany

[9]Rijkswaterstaat, Ministry of Infrastructure and Water Management, Zuiderwagenplein 2, 8224 AD Lelystad, The Netherlands

[10]TNO, Netherlands Organisation for Applied Scientific Research, Princetonlaan 6, 3584 CB Utrecht, The Netherlands

[11]ECSIN-European Center for the Sustainable Impact of Nanotechnology - EcamRicert SRL, C.so Stati Uniti 4, 35127 Padova, Italy

[12]European Commission - Joint Research Centre, via E. Fermi, 2749, 21027 Ispra VA, Italy

[13]Bundesanstalt für Gewässerkunde, Am Mainzer Tor 1, 56068 Koblenz, Germany

[14]Frontier Laboratories Europe, Bandstrasse 39B, 45359 Essen, Germany

[15]Frontier Laboratories Ltd. 4-16-20, Saikon, Koriyama, Fukushima, 963-8862 Japan

[16]Umweltbundesamt, Corrensplatz 1, 14195 Berlin, Germany

[17]Helmholtz Centre for Environmental Research – UFZ, Department of Environmental Analytical Chemistry, Permoserstrasse 15, 04318 Leipzig, Germany

[18]Helmholtz-Zentrum Hereon, Institute of Coastal Environmental Chemistry, Department for Inorganic Environmental Chemistry, Max-Planck-Straße 1, 21502, Geesthacht, Germany

[19]Hohenstein Laboratories GmbH & Co. KG, Schlosssteige 1, 74357 Boennigheim, Germany

[20]Department of Marine Science, College of Natural Sciences, Incheon National University, 119 Academy-ro, Yeonsu-gu, Incheon 22012, Republic of Korea

[21]Institute for Energy and Environmental Technology e.V., Bliersheimer Str. 58 - 60, 47229 Duisburg, Germany

[22]RheinMain University of Applied Sciences, Faculty of Engineering, Institute for Environmental and Process Engineering, Am Brückweg 26, 65248 Rüsselsheim, Germany

[23]Korea Institute of Analytical Science and Technology, SeoulSup AK Valley, Seongsuil-ro 99, Seongdong-gu, Seoul 04790, Republic of Korea

[24]Ecological Risk Research Division, South Sea Research Institute (SSRI), Korea Institute of Ocean Science and Technology (KIOST), 41 Jangmok-1Gi, Jangmok-myon, Geoje-Shi 656-834, Republic of Korea

[25]Department of Civil Engineering, Seoul National University of Science and Technology, Seoul 01811, Republic of Korea

[26]K-water, Sintanjin-ro 200, Daedeok-gu, 34350 Daejeon, Republic of Korea

[27]Labor IBEN GmbH, Am Lunedeich 157, 27572 Bremerhaven, Germany

[28]Leibniz Institute of Photonic Technology e.V. (IPHT), Albert-Einstein-Straße 9, 07745 Jena, Germany

[29]Max Rubner-Institut, Federal Research Institute of Nutrition and Food, Department of Safety and Quality of Milk and Fish Products, Hermann-Weigmann-Straße 1, 24103 Kiel, Germany

[30]Metrology Research Centre, National Research Council Canada, 100 Sussex Drive, Ottawa, ON K1A 0R6, Canada

[31]Laboratoire Softmat, Université de Toulouse, CNRS UMR 5623, Bâtiment 2R1, 118 route de Narbonne 31062 Toulouse cedex 9 France

[32]National Institute for Metrological Research, Strada delle Cacce, 91 10135 Torino, Italy

[33]Marine Environment Research Division, National Institute of Fisheries Science, Busan 46083, Republic of Korea

[34]National Metrology Institute of South Africa (NMISA), Private Bag X34, Lynnwood Ridge, Pretoria 0040, South Africa

[35]The Dove Marine Laboratory, Newcastle University, Newcastle upon Tyne, NE1 7RU, United Kingdom

[36]NILU, Hjalmar Johansens gate 14, 9007 Tromsø, Norway

[37]Climate and Environment dep., Norwegian Research Centre, Mekjarvik 12, 4072 Randaberg, Norway

[38]Laboratoire Phytocontrol - 180 rue Philippe Maupas, 30035 Nîmes, Francehytocontrol, Nîmes, France

[39]Plastics Institute for medium-sized businesses, Karolinenstraße 8, 58507 Lüdenscheid, Germany

[40]Empresa Portuguesa das Águas Livres, S.A. – EPAL, Direção de Laboratórios, 1800-031 Lisboa, Portugal

[41]Private Diepholz University of Economics and Technology, Am Campus 2, 49356 Diepholz, Germany

[42]SGS INSTITUT FRESENIUS GmbH, Königsbrücker Landstraße 161, 01109 Dresden, Germany

[43]SGS Testing & Control Services Singapore Pte Ltd – 30 Boon Lay Way #03-01 Singapore 609957, Singapore

[44]Technical center for plastics processing in France, Biopôle Clermont-Limagne, 3 Rue Emile Duclaux, 63360 Saint-Beauzire, France

[45]PiCA Prüfinstitut Chemische Analytik GmbH, Rudower Chaussee 29, 12489 Berlin, Germany

[46]United Kingdom Centre for Ecology and Hydrology, Wallingford, Oxfordshire OX10 8BB, United Kingdom

[47]Group of Applied Analytical Chemistry, Institute of Environmental Sciences (IUMA), Faculty of Sciences, University of A Coruña, Campus da Zapateira, 15071, A Coruña, Spain

[48]Animal Ecology I and BayCEER, University of Bayreuth, Universitätsstraße 30, 95445 Bayreuth, Germany

[49]University of Belgrade-Faculty of Chemistry, Studentski trg 16, 11000 Belgrade, Serbia

[50]Department of Chemistry "Giacomo Ciamician", University of Bologna, Technopole of Rimini, via Dario Campana 71, 47922 Rimini, Italy

[51]University of Brescia, Department of Mechanical and Industrial Engineering & INSTM RU of Brescia, via Branze 38 25123, Brescia, Italy

[52]University of Oldenburg, Institute for Chemistry and Biology of the Marine Environment, Carl-von-Ossietzky-Straße 9-11, 26129 Oldenburg, Germany

[53]Department of Chemistry and Industrial Chemistry, University of Pisa, via G. Moruzzi 13, 56124, Pisa, Italy

[54]Queensland Alliance for Environmental Health Sciences (QAEHS), The University of Queensland, 20 Cornwall Street, Woolloongabba 4102, QLD, Australia

[55]Wageningen Food Safety Research (WFSR), part of Wageningen University & Research, 6708 WB Wageningen, The Netherlands

[56]State Key Laboratory of Environmental Criteria and Risk Assessment, Chinese Research Academy of Environmental Sciences, No.8, Dayangfang, Beiyuan,Beijing 100012, China

Table of contents

Table S1. Overview of interlaboratory comparisons related to the analysis of microplastic… … … … … … … … … … … S4
Table S2. Mass fraction (as µg polymer per mg tablet) (n=19) in the homogeneity study… … … … … … … … … … … … … ….S5
Table S3. Particle number for both polymers measured with µ-FTIR (n=10)… … … … … … … … … … … … … … … … … … …S5
Table S4. Particle number for both polymers measured with µ-Raman (n=10)… … … … … … … … … … … … … … … … … ….S5
Table S5. Particle number concentration (n=20) per tablet measured by manual counting from SEM micrographs… … … …S5
Figure S1. Overview of participant by country… …..S6
Figure S2. Particle size distribution curves of microplastic polymers as determined with laser diffraction method… … … …S6
Figure S3. Production process of microplastic powder from commercial plastic granulate… … … … … … … … … … … … ….S6
Figure S4. Preparation of the tablets from microplastic powder… …S6
Figure S5. Filtration for thermo-analytical experiments with use of a crucible… … … … … … … … … … … … … … … … …S6
Figure S6. Normalized aged PE particle numbers (> 500 µm) per tablet derived from µ-FTIR, µ-Raman and LDIR… … ….S6
Figure S7. Normalized PET particle numbers (> 500 µm) per tablet derived from µ-FTIR, µ-Raman and LDIR… … … …..S6
Figure S8. Normalized aged PE particle numbers (100 - 500 µm) per tablet derived from µ-FTIR, µ-Raman and LDIR… …S6
Figure S9. Normalized PET particle numbers (100 - 500 µm) per tablet derived from µ-FTIR, µ-Raman and LDIR… … …S6
Figure S10. Normalized aged PE particle numbers (50 - 100 µm) per tablet derived from µ-FTIR, µ-Raman and LDIR…..S7
Figure S11. Normalized PET particle numbers (50 - 100 µm) per tablet derived from µ-FTIR, µ-Raman and LDIR… … …S7
Figure S12. Normalized aged PE particle numbers (10 – 50 µm) per tablet derived from µ-Raman measurement… … … …S7
Figure S13. Normalized PET particle numbers (10 – 50 µm) per tablet derived from µ-Raman measurement… … … … … …S7
Figure S14. Normalized aged PE particle numbers (10 – 50 µm and 20 - 50 µm) per tablet derived from µ-FTIR, LDIR….S7
Figure S15. Normalized PET particle numbers (10 – 50 µm and 20 - 50 µm) per tablet derived from µ-FTIR and LDIR….S7
Figure S16. Normalized aged PE particle numbers (5 – 10 µm) per tablet derived from µ-Raman measurements… … … ….S7
Figure S17. Normalized PET particle numbers (5 – 10 µm) per tablet derived from µ-Raman measurements… … … … … …S7
Table S6. Main sources of uncertainty… …S8
Figure S18. Particle size distribution for PET and aged PE calculated from results derived obtained from SEM… … … … …S8
Table S7. Type of particle counting (automated *vs* manual)… …S9
Recommendations for best practices… ……..S10
References… ……..S10

Table S1. Overview of interlaboratory comparisons related to the analysis of microplastic.

Parameters	Polymers	Particle size (μm)	Matrix	Methods	Nr. labs	Drawbacks	Reference
Particle number, PSD	PP, HDPE, LDPE	400-5700	Seawater	FTIR, μ-FTIR, Raman	12	Polymer identity missing, only large particles	Isobe *et al.*[1]
Mass fraction	PE, PET, PS, PP	145-174	Freshwater sediment	Py-Gc/MS, TED-GC/MS, TGA-FTIR, TGA-MS, DSC,	16	Pristine polymers, narrow particle size range	Becker *et al.*[2]
Particle number, polymer type, or particle mass	PE, PVC, PMMA, PET, PS,	8-140	Ultrapure water	Microscopy, μ-FTIR, μ-Raman spectroscopy, TED-GC/MS, SEM	17	Pristine MP, test material RSD 26-85%, ultra-pure water	Müller *et al.*[3]
Polymer type, particle number, polymer mass	PC, PS, PP, PET, LDPE, EPS	150-300 and 2000-4000	Soda tablets	Microscopy, gravimetric, ATR-FTIR, μFTIR, Py-GC–MS, Raman, μ-Raman spectroscopy.	34	Only large particles	WEPAL-QUASIMEME/NORMAN *et al.*[4]
Polymer type, particle number	PVC, PET, PE, EPS, PS	1-500	Drinking water + gelatine	FTIR, Raman	12		Munno *et al.*[5]
Polymer type, particle number, particle size	PE, PS, PVC, PET	1-500	Drinking water	OM, μ-FTIR, μ-Raman spectroscopy	22	Clean water as matrix	De Frond *et al.*[6]
Particle number, size, mass fraction	PET in water	30-200	Clean water	μ-FTIR, μ-Raman spectroscopy, Py-GC/MS, fluorescence microscopy, TGA, LDIR, NMR, HPLC	98	Clean water as matrix	European Commision[7]
Polymer type, polymer mass	PE, PS, PVC	125-355	Soda tablets,	μ-FTIR, Py-GC/MS, microscopy, μ-Raman spectroscopy, LDIR	7	Narrow particle size range	INOPOL/SINOPLAST[8]
Polymer identity, PSD, particle number	PE, PET, PS, PC, PP, PVC	50-300	Soda tablets, sediments, sand samples	OM, gravimetric, ATR-FTIR, μFTIR, μFTIR-FPA, Py-GC–MS, Raman, μ-Raman spectroscopy, LDIR, Fluorescent	90		EUROqCHARM[9]

				microscopy, stereomicroscopy, SPERO-QT			
Polymer identity, particle count, total weight	HDPE, PET, PC, PP, PVC	300-5000	Marine sediment and sea water	ATR-FTIR, FTIR	5	Large MPs, few laboratories, only two methods	Cadiou et al.[10]

Table S2. Mass fraction (as µg polymer per mg tablet) (n=19) in the homogeneity study

	PET	SD	RSD	PE	SD	RSD
TGA	2.2	0.3	14.1	0.9	0.1	16.0
TED-GC/MS	1.9	0.5	27.5	0.8	0.1	18.0

Table S3. Particle number for both polymers measured with µ-FTIR (n=10) NQAC Vittel

µ-FTIR	PET (n=10)	SD	RSD	PE (n=10)	SD	RSD
>500 µm	0.7	1.6	228.5	3.5	4.9	140.0
100-500 µm	136.5	40.4	29.6	137.8	46.2	33.5
50-100 µm	214.3	45.8	21.3	121.5	38.2	31.4
*20-50 µm	198.4	55.5	27.9	72.9	45.5	62.4
Total	549.9	78.4	14.2	335.7	94.1	28.0

* > 20 µm - lowest limit of confident detection for µ-FTIR

Table S4. Particle number for both polymers measured with µ-Raman (n=10) NQAC Vittel

µ-Raman	PET (n=10)	SD	RSD	PE (n=10)	SD	RSD
> 500 µm	1.0	0.8	80.0	2.2	2.3	104.5
100-500 µm	107.5	15.1	14.0	129.3	25.2	19.4
50-100 µm	265.6	38.4	14.4	210.4	82.8	39.3
20-50 µm	492.6	73.4	14.9	496.9	228.2	45.9
10-20 µm	275.2	33.2	12.0	450.9	193.1	42.8
5-10 µm	152.3	36.7	24.0	266.9	140.1	52.5
Total	1294.2	151.9	11.7	1556.6	631.2	40.5

Table S5. Particle number concentration (n=20) per tablet measured by manual counting from SEM micrographs

	PET (n=10)	SD	RSD	PE (n=10)	SD	RSD
>500 µm	0.0	0.0	0.0	0.1	0.3	244.2
100-500 µm	3.9	2.5	65.4	81.7	30.5	37.4
50-100 µm	171.5	63.2	36.8	298.9	97.8	32.7
10-50 µm	730.3	255.5	34.9	494.7	279.8	56.5
Total	905.7	295.7	32.2	876.8	357.1	40.7

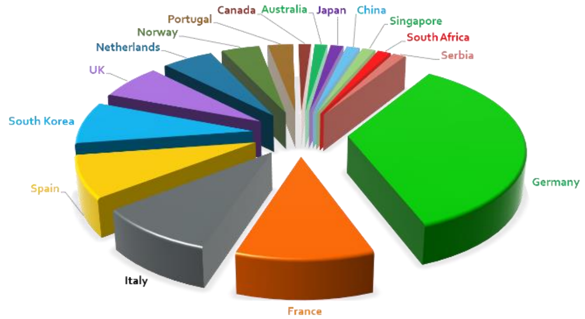

Figure S1. Overview of participants by country

a a b b c

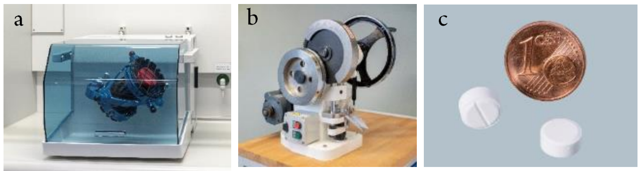

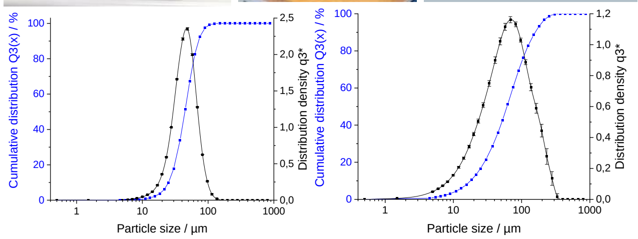

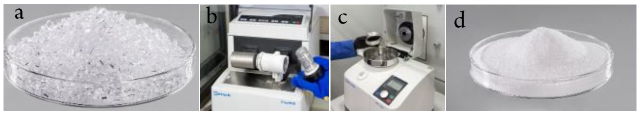

Figure S2. Particle size distribution curves of microplastic polymers as determined with laser diffraction method: a) PET and b) aged PE.

Figure S3. Production process of microplastic powder from commercial plastic granulate: a) plastic granulate, b) cryo-milling, c) sieving, d) microplastic powder (1-100 µm).

Figure S4. Preparation of the tablets from microplastic powder: a) homogenization, b) pressing step with press machine, c) pressed tablets (about 250 mg).

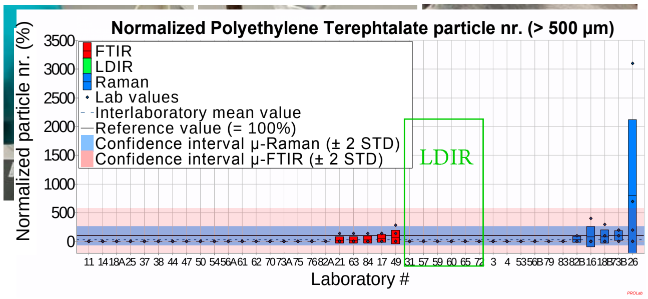

Figure S5. Filtration for thermo-analytical experiments with use of a crucible. a) placing the crucible; b) MilliQ water dropping on the tablet; c) tablet rests (presumably microplastic) after completing the filtration process.

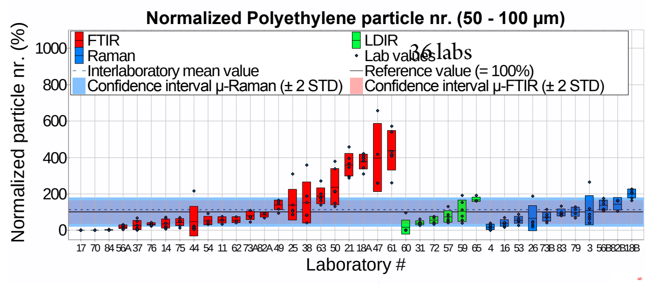

Figure S6. Normalized aged PE particle numbers (> 500 µm) per tablet derived from µ-FTIR, µ-Raman and LDIR measurements. Red, green and blue bars – standard deviations of intra-laboratory results (repeatability).

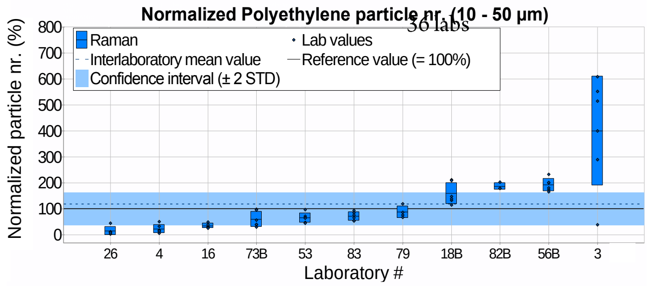

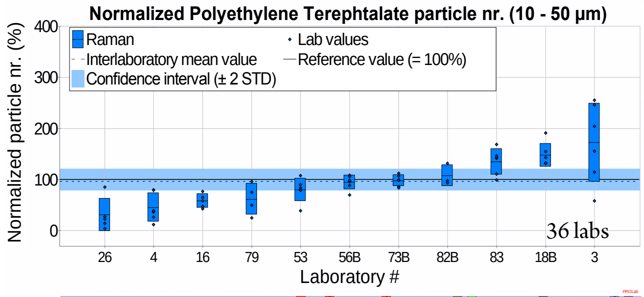

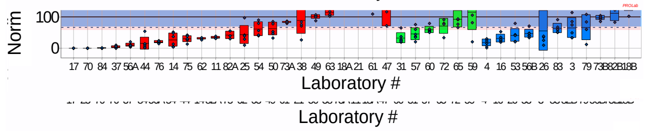

Figure S7. Normalized PET particle numbers (> 500 µm) per tablet derived from µ-FTIR, µ-Raman and LDIR measurements. Red, green and blue bars – standard deviations of intra-laboratory results (repeatability).

Figure S8. Normalized aged PE particle numbers (100 - 500 µm) per tablet derived from µ-FTIR, µ-Raman and LDIR measurements. Red, light green and blue bars – standard deviations of intra-laboratory results (repeatability).

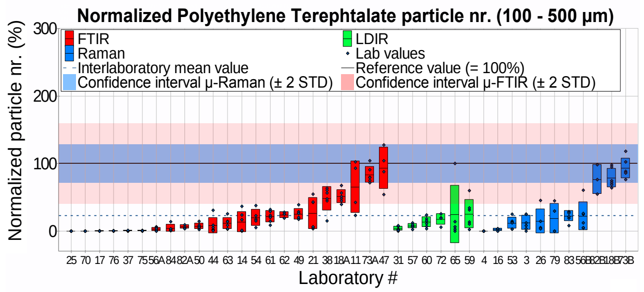

Figure S9. Normalized PET particle numbers (100 - 500 µm) per tablet derived from µ-FTIR, µ-Raman and LDIR measurements. Red, light

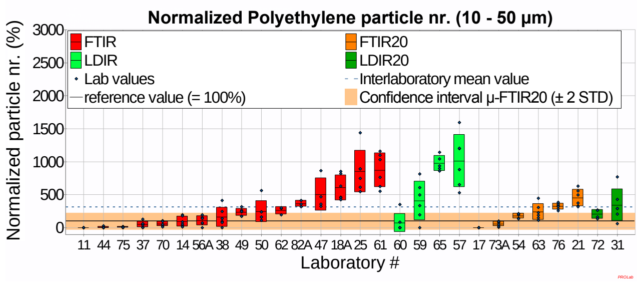

green and blue bars – standard deviations of intra-laboratory results (repeatability).

Figure S10. Normalized aged PE particle numbers (50 - 100 µm) per tablet derived from µ-FTIR, µ-Raman and LDIR measurements. Red, light green and blue bars – standard deviations of intra-laboratory results (repeatability).

36 labs

Figure S11. Normalized PET particle numbers (50 - 100 µm) per tablet derived from µ-FTIR, µ-Raman and LDIR measurements. Red, light green and blue bars – standard deviations of intra-laboratory results (repeatability).

Figure S12. Normalized aged PE particle numbers (10 – 50 µm) per tablet derived from µ-Raman measurements. Blue bars – standard deviations of intra-laboratory results (repeatability).

Figure S13. Normalized PET particle numbers (10 – 50 µm) per tablet derived from µ-Raman measurements. Blue bars – standard deviations of intra-laboratory results (repeatability).

11 labs
36 labs

11 labs

Figure S14. Normalized aged PE particle numbers (10 – 50 µm and [illegible] - 50 µm) per tablet derived from µ-FTIR and LDIR measurements. Red, light green, dark green and orange bars – standard deviations of intra-laboratory results (repeatability).

28 labs

Figure S15. Normalized PET particle numbers (10 – 50 µm and 20 - 50 µm) per tablet derived from µ-FTIR and LDIR measurements. Red, light green, dark green and orange bars – standard deviations of intra-laboratory results (repeatability).

Figure S16. Normalized aged PE particle numbers (5 – 10 µm) per tablet derived from µ-Raman measurements. Blue bars – standard deviations of intra-laboratory results (repeatability).

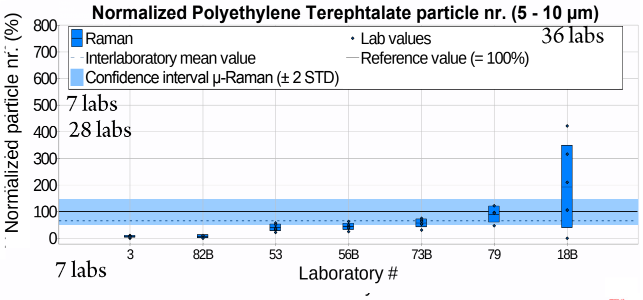

7 labs

Figure S17. Normalized PET particle numbers (5 – 10 µm) per tablet derived from µ-Raman measurements. Blue bars – standard deviations of intra-laboratory results (repeatability).

Table S6. Main sources of uncertainty

Source	Uncertainty	Comment
Sample homogeneity	High	MP content: RSD particle number = 12-40 %, RSD mass fraction = 18-27%
Tablet mass	Low	Tablet mass variation ±5 mg, corresponding to ±2%
Sample preparation (Filtration)	High	Loss of particles during filtration: assumption of minus 10-30%
Method sensitivity	Low-Middle	Determines whether the smallest particles are counted or not
Instrument calibration	Middle-High	Calibration plays a role in trueness/bias of the measurement result. Not provided by the labs
Spectral library used	Middle	There may be differences in matches between a curated vs laboratory generated library
Measurement repeatability	Middle-High	The repeatability of the results based on 6 samples of the same material
Instrument measurement uncertainty	Low	Measurement uncertainty of the measurand measured with the instrument at best calibration and best settings
Instrumental settings	Middle-High	Software parameter settings, like threshold size, threshold to positively identify spectra by comparison against spectral databases, region of interest, aperture, focus, acquisition time, number of pixels, settings stability during measurement, etc.
Background (laboratory and blank samples)	Low	Presence of MPs even in blank samples (negative control). Estimated <1-6%. Laboratory background contamination not assessed
Operator's effect	Middle-High	Untraceable. Explains how accurate the sample is handled and how is the measurement carried out
Extrapolation of results	Middle-High	Analysing only X% of the filter surface (sometimes as low as just 1% of the filter) and extrapolating the result to 100% could alter the particle number.
Accuracy of the counting software	Middle	When using automated particle counting, exact uncertainty unknown, particularly for agglomerated and/or small particles with complex shapes

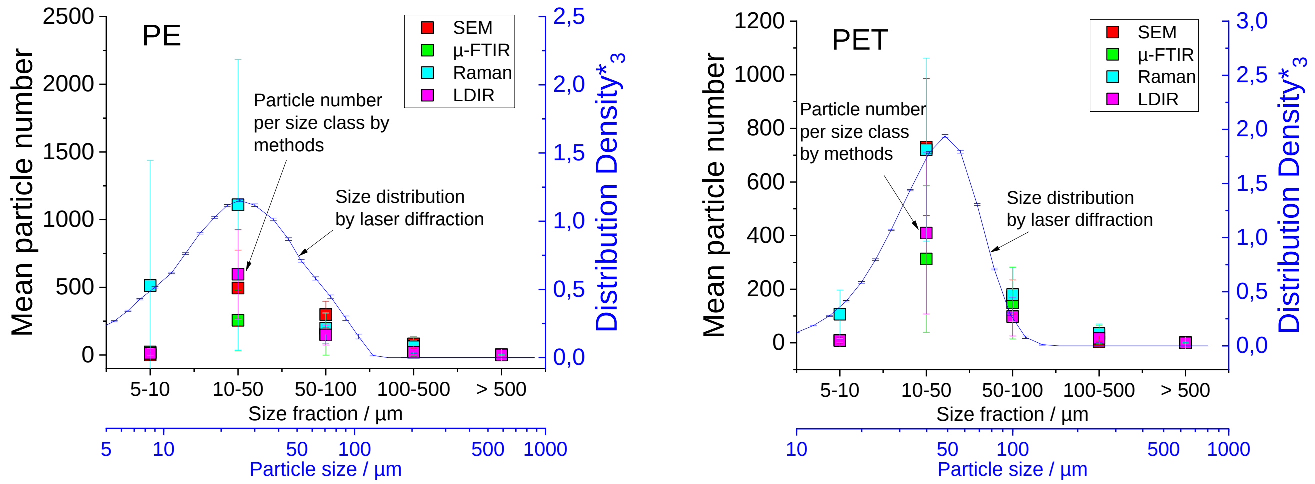

Figure S18. Particle size distribution for aged PE and PET calculated from results derived from SEM, µ-Raman, µ-FTIR, LDIR measurements.

Table S7. Type of particle counting (automated *vs* manual)

Lab Nr.	Method	Automated	Semi-automated	Manually
56	FTIR		YES	
21	FTIR			YES
18	FTIR	YES		
17	FTIR			YES
50	FTIR	YES		
73	FTIR	YES		
3	FTIR	YES		
38	FTIR	YES		
14	FTIR	YES		
82	FTIR	YES		
75	FTIR	YES		
54	FTIR	YES		
47	FTIR	YES		
44	FTIR	YES		
63	FTIR			YES
27	FTIR	YES		
49	FTIR	YES		
84	FTIR	YES		
31	LDIR	YES		
59	LDIR	YES		
72	LDIR	YES		
56	Raman	YES		
18	Raman	YES		
83	Raman	YES		
73	Raman	YES		
3	Raman	YES		
16	Raman			YES
26	Raman	YES		
82	Raman	YES		

Recommendations for best practices:

1. Blank samples

We recommend investigating background contamination in the laboratories as lab blank prior to analysis of the microplastic particles. Specific measures should be undertaken to minimize the contribution of such background contamination to ensure as clean working environment as possible.

2. Filtration of tablets

The filtration step represents a critical step. We recommend here to use the same filter material and pore size as the reference material provider used for determining the reference value. We recommend placing the tablet directly on the filter and rinsing water over the tablet during filtration. If this is not feasible, we recommend dissolving tablets in warm water with ultra sonic bath as long as necessary, until the tablet is dissolved completely before the solution is applied to the filter. We also recommend rinsing with MilliQ water all the walls of the filtration funnel/beaker etc. to transfer the particles quantitively on the filter. Loaded filters should be dried before measurement.

3. Instrumental setting

We noticed that different laboratories used different instrumental settings, probably driven by the experience of individual experts with the own instrument. However, it would be helpful, if all users of the same instrument type (either μ-FTIR or μ-Raman) use the same instrumental settings, to minimize uncertainty contributions derived from different settings. Reference material providers should give detailed setting instructions, e.g. HQI threshold. We recommend use of the same library of spectral lines. This might be discussed in a prior online meeting with participants towards agreement between all the participants. Use of different marker libraries might skew results. We recommend using the same evaluation software; however, the assumption is that the contribution to overall uncertainty due to different software used is rather small.

4. Instrumental calibration

We recommend calibration of the instruments with certified reference materials prior to measurements on microplastic samples.

5. Measurement repeatability

We recommend measurement of tablets possibly on the same day, independent of method. If the material is to be used as RM for method validation, then we would recommend, for example, always taking 3 measurements for each control measurement, e.g. once a month (or once per week).

6. Operators' degree of expertise

We recommend employing most experienced researchers for carrying out experiments. The person carrying out the work should be regularly informed and sensitized about the possibility of contamination.

7. Extrapolation of results

This is probably one of the largest sources of the erroneous measurements. We strongly recommend measuring the whole filter and to not extrapolate results. For example, if 1-2% of the filter is measured, assuming a homogeneous distribution of particles on the whole filter and extrapolating the particles number to 100% of the filter surface area might skew results tremendously. If measuring of the 100% area is not feasible, we recommend taking at least 10 smaller areas at different locations, possibly comprising areas with fewer particles and those with higher particle numbers, so that the overall result is as close as possible to the real particle number.

REFERENCES

(1) Isobe, A.; Buenaventura, N. T.; Chastain, S.; Chavanich, S.; Cózar, A.; DeLorenzo, M.; Hagmann, P.; Hinata, H.; Kozlovskii, N.; Lusher, A. L. An interlaboratory comparison exercise for the determination of microplastics in standard sample bottles. *Marine pollution bulletin* **2019**, *146*, 831-837.

(2) Becker, R.; Altmann, K.; Sommerfeld, T.; Braun, U. Quantification of microplastics in a freshwater suspended organic matter using different thermoanalytical methods – outcome of an interlaboratory comparison. *Journal of Analytical and Applied Pyrolysis* **2020**, *148*, 104829.

(3) Müller, Y. K.; Wernicke, T.; Pittroff, M.; Witzig, C. S.; Storck, F. R.; Klinger, J.; Zumbülte, N. Microplastic analysis—are we measuring the same? Results on the first global comparative study for microplastic analysis in a water sample. *Analytical and bioanalytical chemistry* **2020**, *412* (3), 555-560.

(4) van Mourik, L. M.; Crum, S.; Martinez-Frances, E.; van Bavel, B.; Leslie, H. A.; de Boer, J.; Cofino, W. P. Results of WEPAL-QUASIMEME/NORMANs first global interlaboratory study on microplastics reveal urgent need for harmonization. *Science of The Total Environment* **2021**, *772*, 145071.

(5) Munno, K.; Lusher, A. L.; Minor, E. C.; Gray, A.; Ho, K.; Hankett, J.; T Lee, C.-F.; Primpke, S.; McNeish, R. E.; Wong, C. S.; et al. Patterns of microparticles in blank samples: A study to inform best practices for microplastic analysis. *Chemosphere* **2023**, *333*, 138883.

(6) De Frond, H.; Thornton Hampton, L.; Kotar, S.; Gesulga, K.; Matuch, C.; Lao, W.; Weisberg, S. B.; Wong, C. S.; Rochman, C. M. Monitoring microplastics in drinking water: An interlaboratory study to inform effective methods for quantifying and characterizing microplastics. *Chemosphere* **2022**, *298*, 134282.

(7) Commission, E.; Centre, J. R.; Ramaye, Y.; Stroka, J.; Cella, C.; Held, A.; Robouch, P.; La Spina, R.; Sirio Fumagalli, F.; Méhn, D.; et al. *Current status of the quantification of microplastics in water – Results of a JRC/BAM interlaboratory comparison study on PET in water*; Publications Office, 2021. DOI: doi/10.2760/27641.

(8) Martínez-Francés, E.; van Bavel, B.; Hurley, R.; Nizzetto, L.; Pakhomova, S.; Buenaventura, N. T.; Singdahl-Larsen, C.; Magni, M.-L. T.; Johansen, J. E.; Lusher, A. Innovative reference materials for method validation in microplastic analysis including interlaboratory comparison exercises. *Analytical and Bioanalytical Chemistry* **2023**, *415* (15), 2907-2919.

(9) *Analysis of microplastics in environmental matrices: results of the interlaboratory comparison study.* https://www.euroqcharm.eu/en/news/analysis-of-microplastics-in-environmental-matrices-results-of-the-interlaboratory-comparison-study (accessed.

(10) Cadiou, J. F.; Gerigny, O.; Koren, Š.; Zeri, C.; Kaberi, H.; Alomar, C.; Panti, C.; Fossi, M. C.; Adamopoulou, A.; Digka, N.; et al. Lessons learned from an intercalibration exercise on the quantification and characterisation of microplastic particles in sediment and water samples. *Marine Pollution Bulletin* **2020**, *154*, 111097.
